# An Unusual Pattern of Acromioclavicular Joint Instability With Coracoid Base Fracture

**DOI:** 10.1155/cro/5181452

**Published:** 2026-04-06

**Authors:** Mara Dimitriu, Markus Scheibel, Florian Freislederer

**Affiliations:** ^1^ Department of Shoulder and Elbow Surgery, Schulthess Klinik, Zürich, Switzerland, schulthess-klinik.ch; ^2^ CMSC Center for Musculoskeletal Surgery, Charité-Universitaetsmedizin Berlin, Berlin, Germany, charite.de

**Keywords:** acromioclavicular joint dislocation, case report, coracoid process fracture, dual fixation, hook-plate fixation

## Abstract

**Background:**

Concurrent acromioclavicular (AC) joint dislocation and coracoid process fracture is a rare injury pattern, representing a double disruption of the superior shoulder suspensory complex (SSSC) and resulting in mechanical instability. Due to its rarity, there is no consensus on optimal management.

**Case Presentation:**

A 43‐year‐old male sustained a right shoulder injury during a snowboarding accident. Imaging revealed a right‐sided AC joint dislocation with minimal superior displacement (coracoclavicular [CC] distance 13.6 mm on the right vs. 12 mm contralaterally) and a base‐near coracoid fracture (Ogawa Type I and Eyres Type IV). Clinical examination showed clavicular elevation and tenderness over the AC joint, with preserved neurovascular function. Surgical management included AC joint stabilization with a short hook plate and screw osteosynthesis of the coracoid fracture. Postoperative care involved immobilization in a functional brace with staged passive and active‐assisted range‐of‐motion exercises, progressing to strengthening from Week 12.

**Outcome and Follow‐Up:**

At 6 and 12 weeks, radiographs confirmed maintained reduction and fracture healing. The hook plate was removed 3.5 months postoperatively. Follow‐up 6 weeks later demonstrated a healed coracoid base, congruent AC joint, minimal pain, and good shoulder function.

**Discussion:**

Surgical fixation restores biomechanical integrity of the SSSC and allows early functional rehabilitation. Combined AC and coracoid fixation represent a viable option, though isolated AC stabilization may also yield favorable outcomes if intraoperative imaging confirms adequate indirect reduction of the coracoid fracture. In our opinion, combined fixation provides a very stable construct with the best chances for anatomical healing and restoration of SSSC integrity.

**Conclusion:**

Concurrent AC dislocation and coracoid fracture is a rare injury that requires careful diagnostic assessment, as the coracoid fracture may be overlooked on conventional imaging. This case demonstrates that surgical management with AC stabilization using a hook plate and coracoid screw fixation can achieve excellent radiological and clinical outcomes.

**Learning Points**

• Minimal increase in coracoclavicular (CC) distance (< 25% compared with the contralateral side) does not exclude a concomitant coracoid fracture and represents a diagnostic pitfall.
• Computed tomography should be performed in cases of acromioclavicular (AC) joint dislocation with minimal CC widening when clinical findings appear disproportionate to radiographic displacement or when additional pathologies are suspected on radiographs.
• Intraoperative assessment of fracture configuration and stability is crucial; base‐near fractures with potential rotational instability may warrant additional coracoid fixation rather than isolated hook‐plate stabilization.
• Hook‐plate removal is typically recommended after radiological consolidation, approximately 3–4 months postoperatively.
• Early rehabilitation should focus on protected passive mobilization while avoiding excessive loading until fracture healing is confirmed.

## 1. Introduction

The AC joint dislocation accounts for approximately 12% of all shoulder injuries and represents a common lesion of the shoulder girdle. Typical trauma mechanisms include indirect axial force transmission to the shoulder, such as a fall onto an outstretched arm (e.g., bicycle accident), or direct impact, as seen in contact sports [1, 2]. Coracoid process fractures are uncommon, comprising approximately 3%–13% of scapular fractures, which in turn account for only about 1% of all fractures. They typically occur as a result of high‐energy trauma and are often associated with additional injuries to the shoulder girdle [3, 4]. Concurrent AC joint dislocation and coracoid process fracture is an uncommon injury pattern, with evidence in the literature limited to individual case reports. Given the rarity of this injury pattern, there is no consensus regarding the optimal treatment approach. This injury pattern represents a double disruption of the superior shoulder suspensory complex (SSSC), resulting in construct instability. As such, surgical intervention is considered the treatment of choice in most cases. The SSSC comprises the glenoid process, coracoid process, CC ligaments, distal clavicle, AC joint, coracoacromial ligament, and the acromion. A “double disruption” refers to the involvement of any two of these anatomical structures, leading to a mechanically unstable shoulder girdle [5–7].

We report a rare case of concurrent AC joint dislocation and coracoid process fracture in a 43‐year‐old male patient. He was treated surgically with a hook plate and open reduction and internal fixation (ORIF) of the coracoid fracture. The patient provided written informed consent for publication of this case. This case report was prepared following the CARE guidelines [8]. All the images have patient authorization for publication.

## 2. Case Presentation

### 2.1. Patient Information

A 43‐year‐old male patient presented 2 days after sustaining a shoulder injury during a snowboarding accident, in which he fell directly onto his right shoulder. Initial imaging, performed at a regional hospital near the site of the accident, revealed an AC joint dislocation in combination with a coracoid process fracture. At presentation, the shoulder was immobilized in a functional brace, and the patient reported significant pain. He works in field sales and regularly engages in recreational sports without a specific athletic focus. His medical history is unremarkable apart from obstructive sleep apnea syndrome (OSAS).

### 2.2. Clinical Findings

Clinical examination revealed a visible elevation of the right clavicle. No hematoma was observed, and moderate swelling was noted in comparison to the contralateral side. Palpation over the AC joint elicited marked tenderness even with minimal pressure. Sensory and motor function in the distribution of the axillary and peripheral nerves were preserved.

### 2.3. Diagnostic Assessment

**Figure 1:**
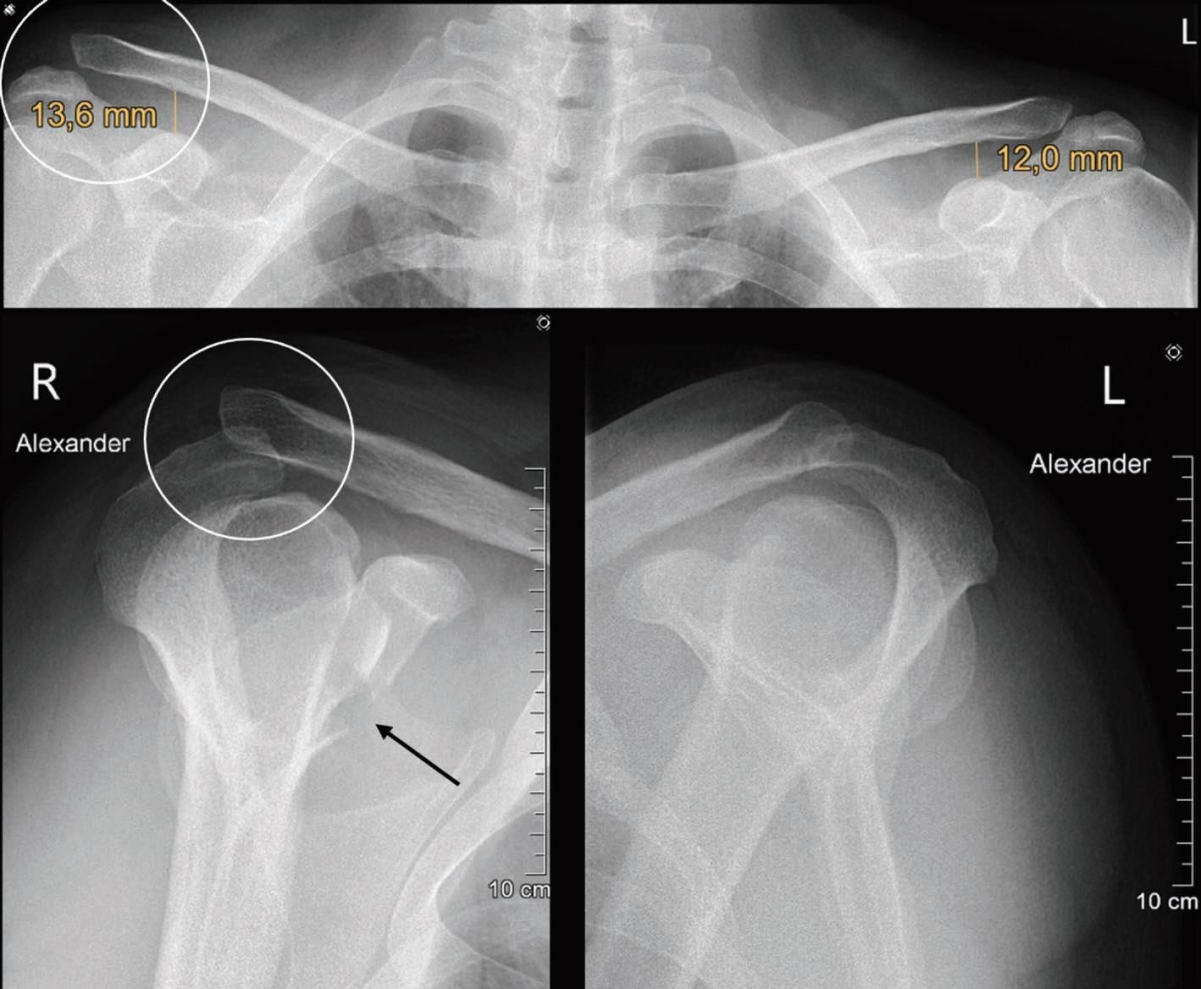
Preoperative panoramic x‐ray (top image) showing right‐sided AC dislocation (circle) with an increased CC distance of 13.6 mm on the right compared with 12 mm on the left. The Alexander view of the right shoulder (bottom‐right image) demonstrates luxation of the clavicle in comparison to the left Alexander view (bottom‐left image, circle) and also reveals the associated fracture of the coracoid process (arrow).

At the initial presentation, bilateral shoulder radiographs, including standard panoramic and Alexander views were performed and revealed a right‐sided AC joint dislocation with superior displacement of the clavicle. In the panorama overview x‐ray of the shoulder girdle, the CC distance measured 13.6 mm on the affected side compared with 12 mm contralaterally, corresponding to an increase of approximately 13% relative to the uninjured side. Based on the slight superior displacement of the distal clavicle on radiographs and the complete rupture of the AC capsule observed intraoperatively, the injury was classified as consistent with a low‐grade Rockwood Type III AC joint dislocation. Posterior displacement of the clavicle was detected. In cases of AC joint dislocation with less than 25% increase in CC distance compared with the contralateral side, a concomitant coracoid fracture should be suspected, particularly when clinical findings appear disproportionate to the degree of radiographic displacement. In such situations, computed tomography is strongly recommended, as coracoid fractures are frequently overlooked on conventional radiographs due to bony overlap. Subsequent computed tomography confirmed a base‐near fracture of the coracoid process (Figure 1).

**Figure 2:**
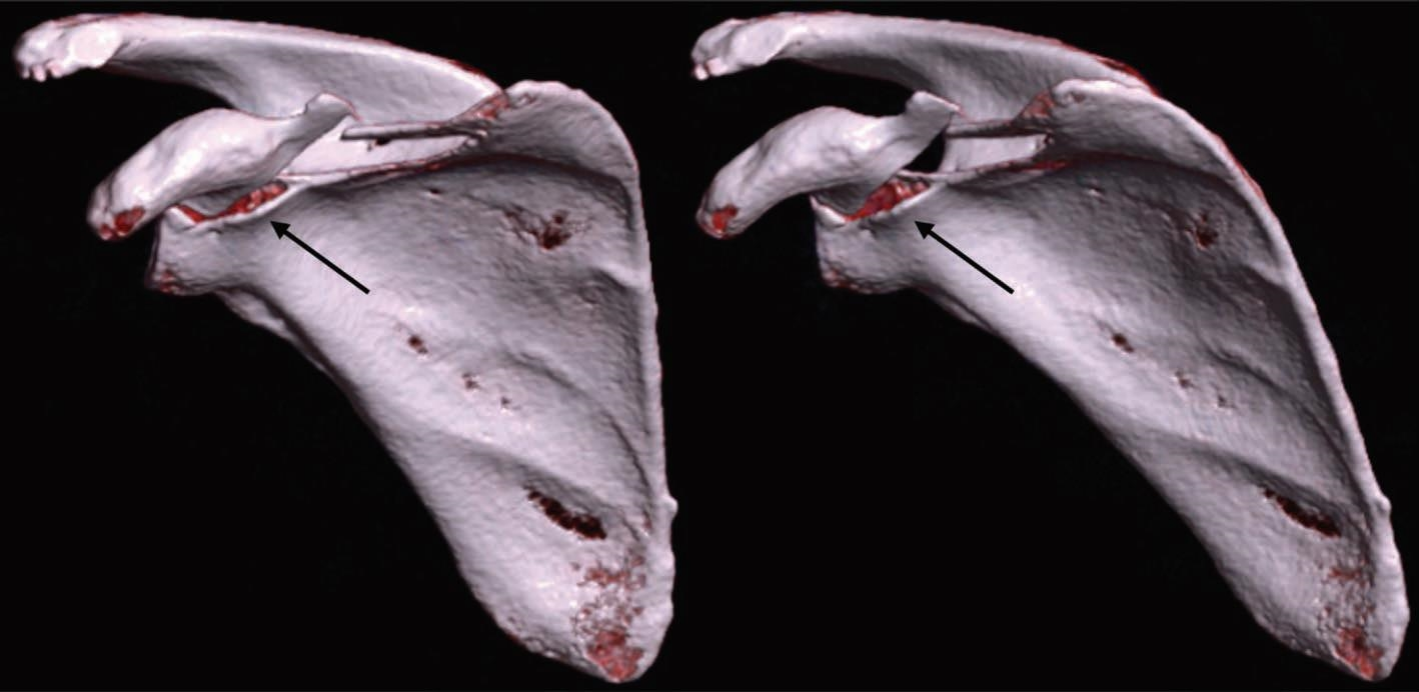
3D sections from the preoperative CT illustrating the base fracture of the coracoid process. Arrows indicate the coracoid base fracture.

A CT scan performed confirmed a base‐near coracoid process fracture classified as Type I according to Ogawa et al. [4] and Type IV according to Eyres et al. [9] and an AC joint dislocation with slight superior displacement of the distal clavicle. (Figure 2).

### 2.4. Therapeutic Intervention

Given the combined injury pattern and the resulting relative instability of the shoulder girdle, we recommended a surgical treatment. Planned management included stabilization of the AC joint using a hook plate, with additional fixation of the coracoid process using screws depending on intraoperative repositioning. Removal of the hook plate was scheduled for approximately 3 months after surgery.

**Figure 3:**
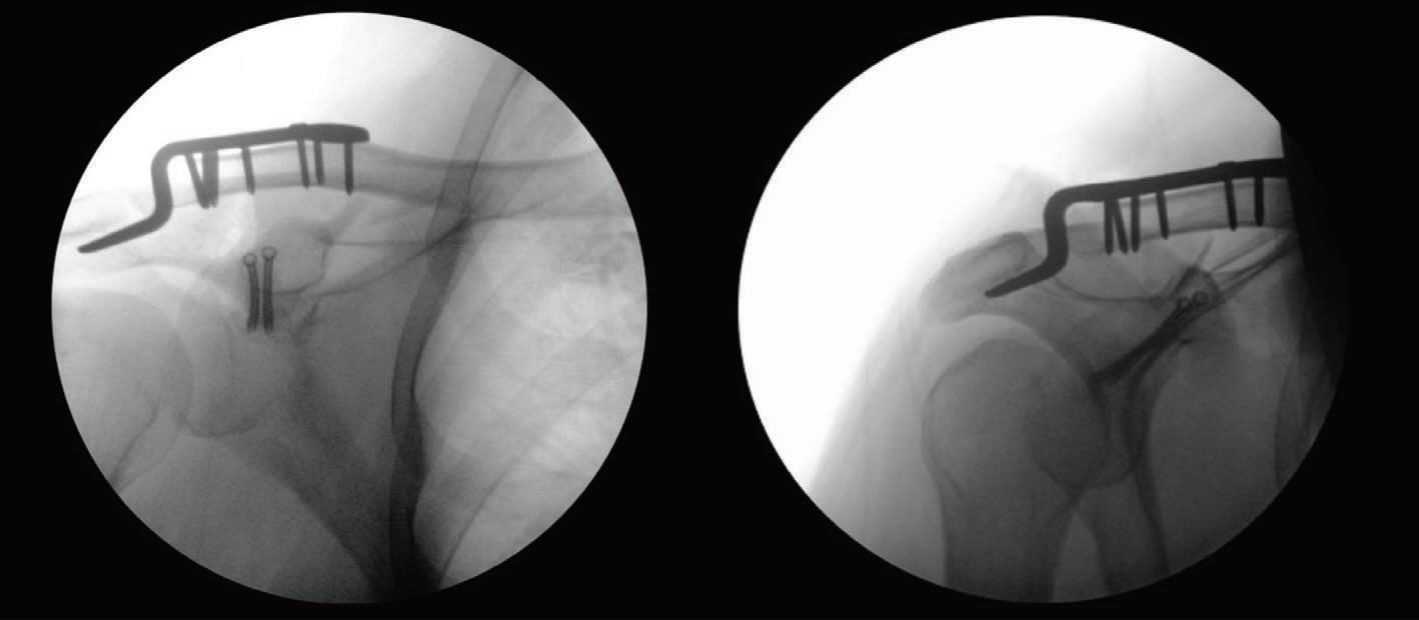
Intraoperative fluoroscopic image: The left image is an AP view, and the right image is an outlet view. Both demonstrate an anatomical reduction of the AC joint, as well as correct positioning and sizing of the hook plate and screws.

The operation was performed 6 days postinjury at the next available surgical slot in the operating schedule. The approach was made via a saber‐cut incision over the lateral clavicle extending to the coracoid process. The exposure revealed a completely torn AC capsule and a dislocated clavicle, which was easily repositioned. Stabilization was achieved by applying a short hook plate, fixed medially with a cortical screw and secured with six locking screws—four lateral and two medial to the cortical screw—providing stable fixation. The coracoid tip was then exposed from below via blunt dissection through the deltoid muscle. Indirect reduction of the coracoid fracture was facilitated by applying upward and medial pressure. Kirschner wires were placed under fluoroscopic guidance in anteroposterior (AP) and outdlet view to temporarily fix the coracoid base, followed by insertion of 3.0‐mm cannulated screws to secure definitive fixation. Fluoroscopy confirmed proper reduction of the AC joint and stable implant positioning (Figure 3). The wound was irrigated and closed in layers. Postoperatively, the arm was immobilized in an Ultrasling Quadrant brace.

The shoulder was immobilized in an Ultrasling Quadrant brace for the initial 6 weeks. Passive range of motion exercises began early, with flexion and abduction limited to 45° and external rotation to 30° during Weeks 1–3, increasing to 60° for each by Week 4. Active‐assisted movements started in Week 5. The internal rotation was allowed up to the abdomen for the first 5 weeks. By Weeks 6–7, flexion and abduction were allowed up to 90°, with free, pain‐free rotation. From Week 8, exercises progressed to full range of motion, followed by strength training from Week 12.

### 2.5. Follow‐Up and Outcome

**Figure 4:**
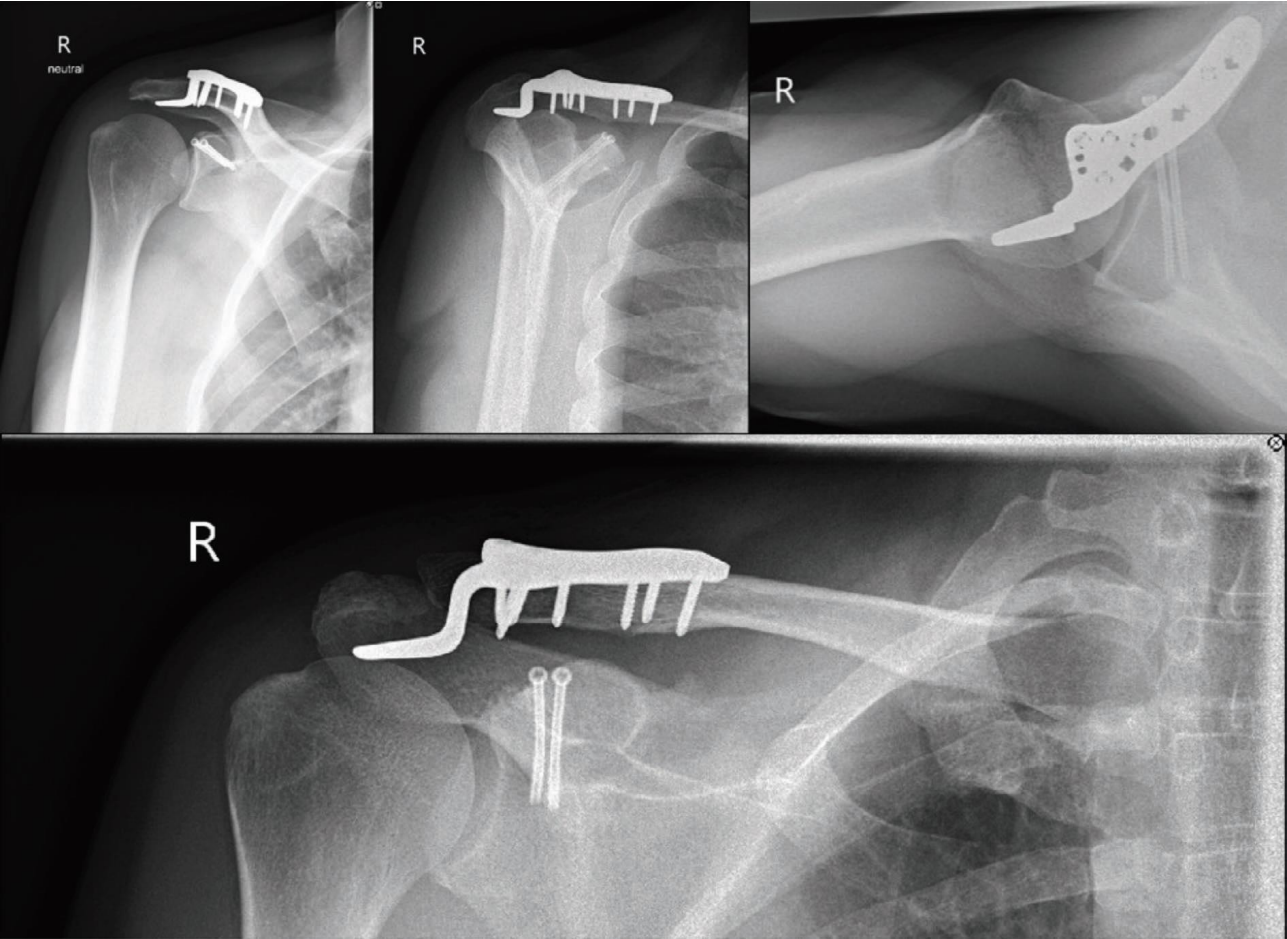
Six‐week follow‐up. Postoperative x‐rays show a good reduction of the AC joint and coracoid fracture. The images are arranged from top left to right as follows: true AP shoulder view, outlet view (top middle), axial view (top right), and Zanca view (bottom).

**Figure 5:**
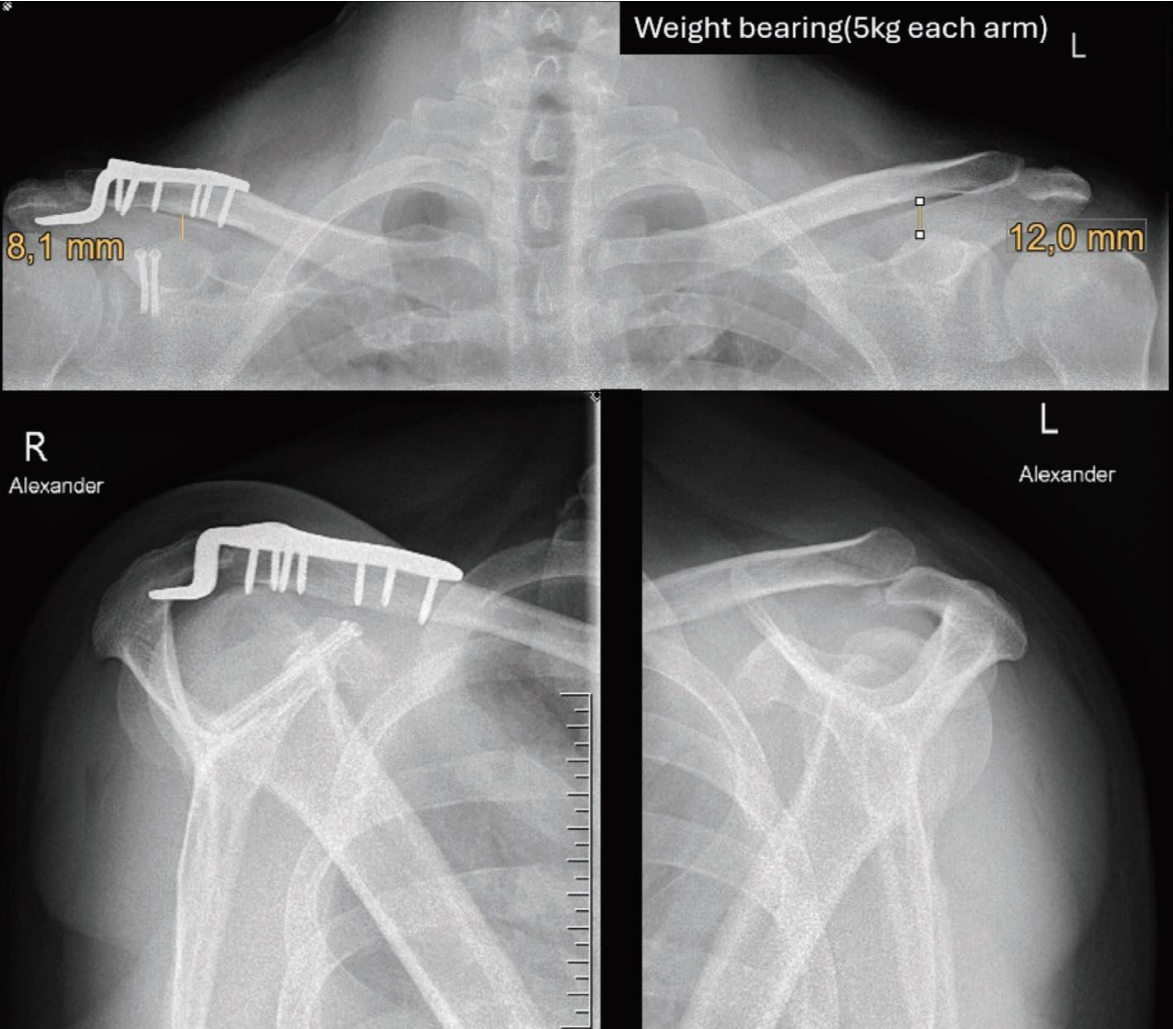
Twelve‐week follow‐up. Postoperative loaded panoramic x‐ray (5 kg per side, top image) showing the correct repositioning of the right AC joint with a CC distance of 8.1 mm. The Alexander view of the right shoulder (bottom‐left image) reveals a physiological alignment of the clavicle in comparison with the left Alexander view (bottom‐right image) and an anatomical repositioning of the coracoid process.

**Figure 6:**
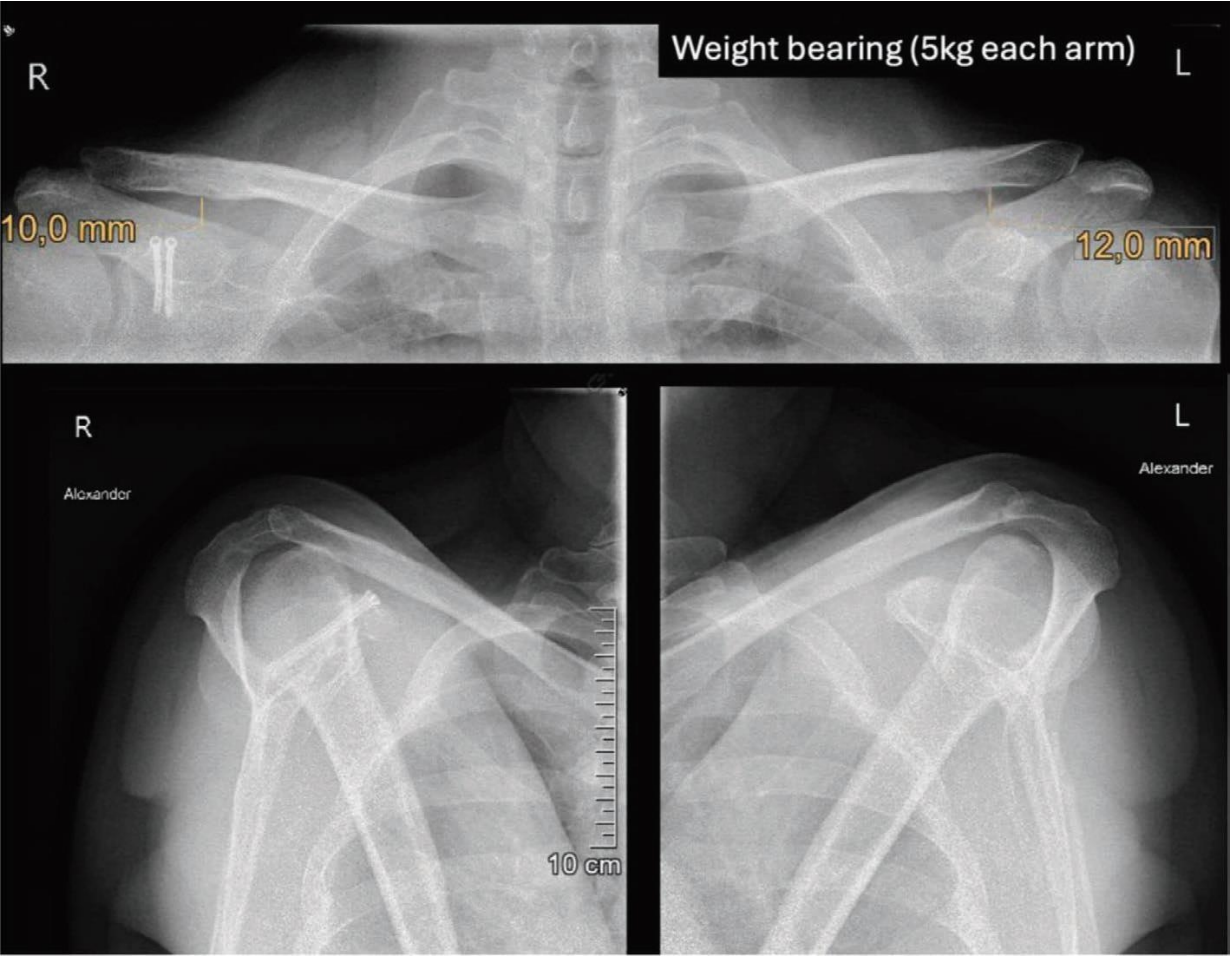
Six‐week follow‐up after hook‐plate removal. The loaded panoramic x‐ray (5 kg per side, top image) shows the preservation of repositioning of the right AC joint with a CC distance of 10 mm. The Alexander view of the right shoulder (bottom‐left image) displays the physiological alignment of the clavicle in comparison to the left Alexander view (bottom‐right image).

**Figure 7:**
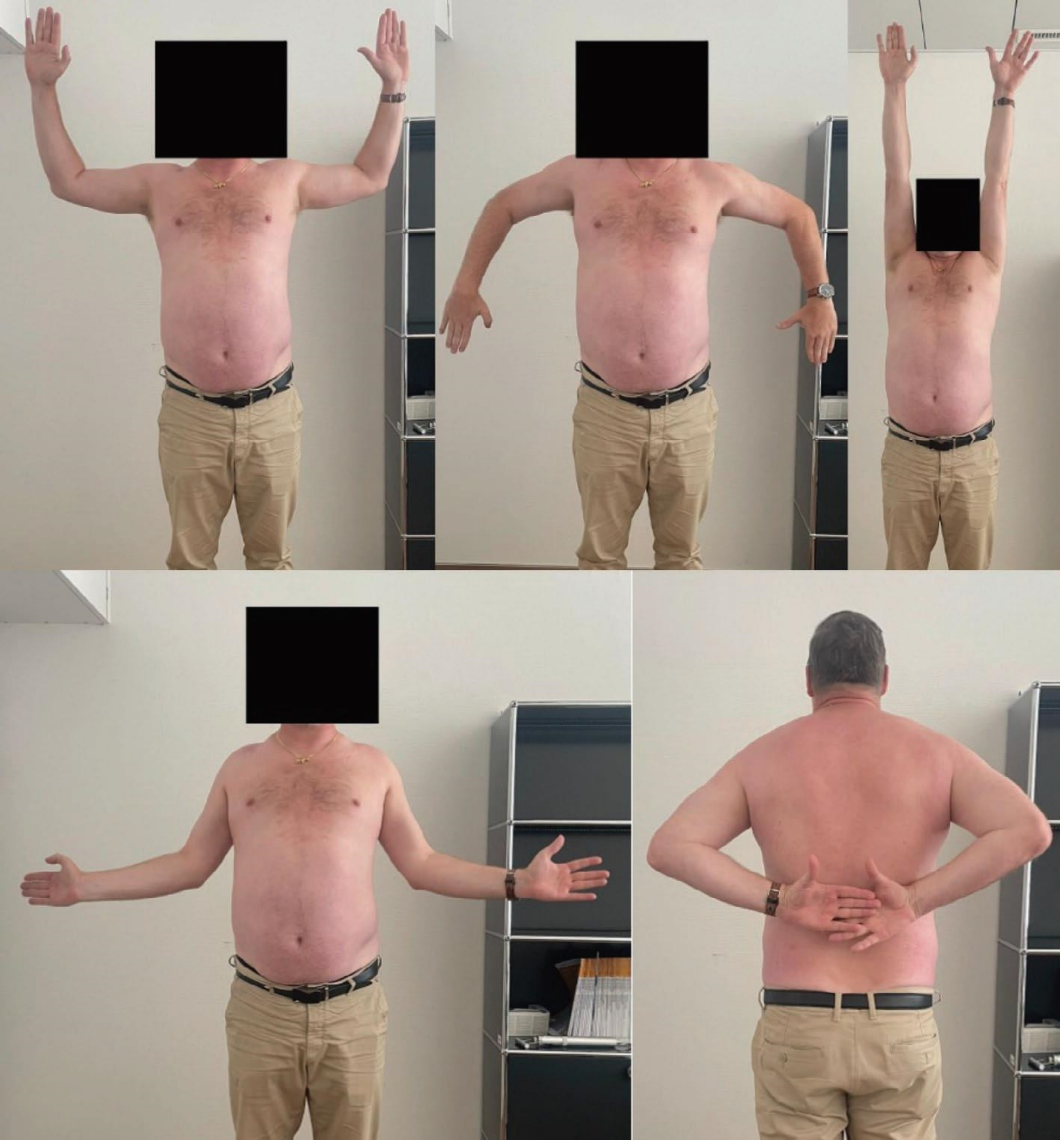
Six weeks following hook‐plate removal, the patient exhibits a shoulder range of motion comparable with the contralateral side, indicating restoration of symmetric mobility.

Postoperative follow‐ups at 6 and 12 weeks demonstrated an uneventful recovery. Radiological imaging confirmed maintained reduction, and the patient reported no significant pain at both visits (Figures 4 and 5). The hook plate was removed 3.5 months postoperatively. At the 6‐week follow‐up after plate removal, the patient showed a favorable functional and radiological outcome with a healed coracoid base in anatomical position, a congruent AC joint and minimal pain and good shoulder function with a constant score of 93. (Figures 6 and 7). At that time point, he had returned to his professional activities in field sales without restrictions. No further activity restrictions were required. The patient progressively resumed recreational sports, reporting no functional limitations or instability. Light daily activities were resumed earlier during the rehabilitation phase (Table 1).

**Table 1:** Timeline of the patient’s clinical course and management.

Time point	Clinical event
Day 0	Snowboarding accident with direct trauma to the right shoulder
Day 2	Presentation at our institution; clinical examination and bilateral shoulder radiographs performed; CT scan confirmed coracoid base fracture (Ogawa Type I, Eyres Type IV) and AC joint dislocation
Day 6	Surgical intervention: AC joint stabilization using hook plate and screw fixation of the coracoid fracture
Weeks 1–3	Immobilization in Ultrasling brace; passive mobilization (flexion/abduction limited to 45°, external rotation to 30°)
Week 4	Increased passive range of motion (flexion/abduction to 60°)
Week 5	Initiation of active‐assisted mobilization
Weeks 6–7	Flexion and abduction permitted up to 90°; pain‐free rotational movements
Week 8	Progression to full range of motion exercises
Week 12	Initiation of strengthening exercises
3.5 months postoperatively	Hook‐plate removal
6 weeks after implant removal	Full range of motion comparable to contralateral side; constant score of 93; radiological confirmation of fracture healing

## 3. Discussion

This case describes a very good functional and radiological outcome after a rare combination of AC joint dislocation and coracoid process fracture, representing a double disruption of the SSSC. This injury pattern is infrequently reported in the literature and presents important considerations regarding diagnosis, classification, and management. The mechanism of injury is frequently described as a high‐energy impact to the affected shoulder, such as occurs in bicycle accidents or motor vehicle collisions, often accompanied by additional injuries. In the present case, the patient sustained a snowboarding accident, which represents a high‐energy trauma consistent with the mechanisms reported in the literature [10–12].

Different theories have been proposed regarding the injury mechanism of this combined pathology. Many authors suggest that it occurs through a mechanism similar to that of isolated AC joint dislocation. Two main trauma patterns are typically discussed in the context of AC joint dislocation: a direct impact to the shoulder and an indirect axial force transmitted through a fall onto the outstretched, adducted arm [13]. The resulting axial traction may lead to rupture of the AC ligaments, whereas instead of tearing the CC ligaments, the force may cause an avulsion fracture of the coracoid process, thus allowing cranial displacement of the clavicle [14–18]. This mechanism has been described, for instance, by Liau et al. in a 53‐year‐old patient [19]. However, the hypothesis that intact CC ligaments cause avulsion of the coracoid process has been challenged by some authors, arguing that this pattern is only plausible in adolescents, where the unfused epiphyseal plate is biomechanically weaker than the ligaments. In adults, the clavicle and coracoid process are generally considered stronger than the CC ligaments [14, 15]. An alternative mechanism, initially proposed by Wilson and Colwill and later supported by others, involves forceful traction by the conjoint tendons—such as during resisted elbow flexion—or by the pectoralis minor, exerting significant tensile force on the coracoid process and resulting in fracture [20, 21]. This explanation, however, primarily applies to tip fractures, whereas most cases involve a fracture at the coracoid base [11, 13, 17, 22]. In agreement with other authors, we believe the most plausible mechanism is a combination of a high‐energy direct impact to the shoulder with simultaneous muscular traction from structures inserting on the coracoid process [11, 12].

For the diagnosis of AC joint dislocation, conventional radiographs—typically a bilateral stress (panorama) view and Alexander view in our clinical practice—are usually sufficient. However, due to bony overlap, coracoid fractures are often missed on standard radiographs; therefore, if such an injury is suspected, additional imaging with computed tomography should be performed [3, 12, 23]. A typical feature of this combined injury pattern is a no or only minimally increased CC distance due to the often intact remaining CC ligaments, which may obscure the diagnosis. Thus, in cases of AC dislocation without relevant CC widening, a concomitant coracoid fracture should be considered and actively sought.

According to the most recent publication known to us, a total of 108 cases of combined AC joint dislocation and coracoid process fracture have been reported in the literature to date, excluding articles published in German and Chinese. Both conservative and surgical treatments have been employed, though more recent reports tend to favor operative management. However, based on systematic reviews from 2023 to 2024, surgical treatment does not appear to offer a clear advantage over conservative approaches [24, 25]. Nonetheless, more robust data and larger patient cohorts are needed to draw definitive conclusions. Conservative management has been associated more frequently with persistent symptoms, including shoulder impingement and bursitis [26], pain at the AC joint [15], and cosmetic deformities [15, 27]. Surgical intervention is commonly indicated due to the mechanical instability resulting from a double disruption of the SSSC. Within the existing literature, several surgical strategies have been described for this combined injury pattern. In particular, Elshahhat and Ahmed recently reported a case series focusing on dual fixation for coracoid base fractures associated with acute AC joint disruption [28]. Their work provides a detailed and technically precise description of the surgical technique, including implant choice and fixation strategy, and demonstrates favorable clinical outcomes. The findings of their series support the biomechanical rationale of combined fixation in selected cases. Our case is consistent with this approach and further illustrates the diagnostic challenges, intraoperative decision‐making, and postoperative radiological and clinical course in an individual patient treated with combined AC and coracoid fixation. Further arguments in favor of surgery include the option for early functional rehabilitation [29] and the avoidance of potential cosmetic deficits associated with nonoperative treatment.

In the present case, we opted for a combined surgical approach involving AC joint stabilization using a hook plate and screw osteosynthesis of the coracoid process. Several case series have also reported successful treatment by stabilizing only the AC dislocation with a hook plate, thereby achieving indirect reduction of the coracoid fracture. This approach has also been applied to Ogawa Type I fractures, for which direct fixation was originally recommended when occurring in isolation [4], and has yielded favorable results [10]. Across all reported cases, where hook plate alone was used, clinical outcomes were good to excellent, and radiological follow‐up consistently demonstrated fracture healing whenever adequate indirect reduction was achieved. Although observed rarely, cases of nonunion where intraoperative reduction was not achieved have been described. Nevertheless, patient‐reported outcomes regarding pain and function remained very good [11]. The main advantages of this technique include preservation of the CC ligaments, which may be compromised during direct coracoid fixation, as well as reduced risk to the adjacent neurovascular structures [10, 11]. A known drawback of hook plates includes mechanical irritation, and less commonly, osteolysis; removal is generally required in a secondary procedure [30, 31].

In the present patient, however, intraoperative assessment revealed a base‐near fracture with potential rotational instability. Although indirect reduction through clavicular stabilization was technically achievable, we considered that hook‐plate fixation alone might not reliably maintain anatomical alignment throughout the healing process. Given the double disruption of the SSSC, restoration of bony continuity at the coracoid base was deemed crucial to re‐establish biomechanical stability. The fracture configuration allowed safe screw placement under direct visualization. Although coracoid fixation carries potential risks due to the proximity of neurovascular structures, including the brachial plexus and suprascapular vessels, careful blunt dissection and fluoroscopic guidance permitted secure implant positioning. After weighing the procedural risks against the expected biomechanical benefits, we concluded that additional screw fixation would provide greater construct stability and optimize the likelihood of anatomical healing in this individual case.

Isolated fixation of the coracoid process has been described only in individual cases, and meaningful conclusions regarding its efficacy cannot be drawn [15, 32–36].

We believe that, in addition to AC joint stabilization, simultaneous osteosynthesis of the coracoid process is an option—provided the surgeon is familiar with the technique—as it restores a very strong fixation and with potentially best chances for anatomical healing and biomechanical integrity of the SSSC. Although long‐term outcomes are not yet available in our case, we consider this combined approach to provide the highest potential for achieving both anatomical healing and excellent functional results.

## 4. Patient Perspective

At final follow‐up, the patient reported high satisfaction with the surgical outcome. He described minimal residual pain and was able to return to his professional activities in field sales without limitations. Recreational sports activities were resumed progressively. He expressed satisfaction with both the functional recovery and the cosmetic appearance of the shoulder.

## 5. Conclusion

The combination of an AC joint dislocation and a coracoid process fracture represents a rare injury pattern that requires careful diagnostic assessment, as the coracoid fracture is easily overlooked on conventional imaging. To date, there is no consensus on the optimal treatment strategy. In the present case report, we demonstrate that surgical management—consisting of AC joint stabilization using a hook plate and screw osteosynthesis of the coracoid process—represents an anatomic and very stable surgical option and results in an excellent radiological and clinical outcome.

## Funding

No funding was received for this manuscript.

## Ethics Statement

This case report was conducted in accordance with institutional guidelines and the principles of the Declaration of Helsinki. According to institutional regulations, formal ethics committee approval was not required for publication of a single case report, provided that written informed consent was obtained.

## Consent

Written informed consent was obtained from the patient for publication of this case report and all accompanying images.

## Conflicts of Interest

The authors declare no conflicts of interest.

## Supporting information


**Supporting Information** Additional supporting information can be found online in the Supporting Information section. Supporting Information: CARE Checklist for Case Reports.

## Data Availability

The data that support the findings of this study are available on request from the corresponding author. The data are not publicly available due to privacy or ethical restrictions.
