# Supplementary material for: An Unusual Pattern of Acromioclavicular Joint Instability With Coracoid Base Fracture
Source: Case Rep Orthop. 2026 Apr 6;2026:5181452. doi: 10.1155/cro/5181452 (PMC13051792; doi:10.1155/cro/5181452)
Supplement: Supplementary file 1 — Supporting Information Additional supporting information can be found online in the Supporting Information section. Supporting Information: CARE Checklist for Case Reports. [file CRO-2026-5181452-s001.pdf]

CARE case report guidelines

CARE Checklist of information to include when writing a case report

(cc) BY-NC-ND

Topic	Item	Checklist item description	Reported on Line
Title	1	The diagnosis or intervention of primary focus followed by the words "case report"	1,2
Key Words	2	2 to 5 key words that identify diagnoses or interventions in this case report, including "case report"	21-23
Abstract (no references)	3a	Introduction: What is unique about this case and what does it add to the scientific literature?	27-30
	3b	Main symptoms and/or important clinical findings	32,35,36
	3c	The main diagnoses, therapeutic interventions, and outcomes	33-35, 37-44
	3d	Conclusion—What is the main "take-away" lesson(s) from this case?	53-56
Introduction	4	One or two paragraphs summarizing why this case is unique (**may include references**)	60-81
Patient Information	5a	De-identified patient specific information.	87, 88
	5b	Primary concerns and symptoms of the patient.	87, 88, 90, 91
	5c	Medical, family, and psycho-social history including relevant genetic information	91-94
	5d	Relevant past interventions with outcomes	Not applicable
Clinical Findings	6	Describe significant physical examination (PE) and important clinical findings.	96-101
Timeline	7	Historical and current information from this episode of care organized as a timeline	204-205
Diagnostic Assessment	8a	Diagnostic testing (such as PE, laboratory testing, imaging, surveys).	104-135
	8b	Diagnostic challenges (such as access to testing, financial, or cultural)	Not applicable
	8c	Diagnosis (including other diagnoses considered)	Not applicable
	8d	Prognosis (such as staging in oncology) where applicable	Not applicable
Therapeutic Intervention	9a	Types of therapeutic intervention (such as pharmacologic, surgical, preventive, self-care)	139-143
	9b	Administration of therapeutic intervention (such as dosage, strength, duration)	144-165
	9c	Changes in therapeutic intervention (with rationale)	Not applicable
Follow-up and Outcomes	10a	Clinician and patient-assessed outcomes (if available)	174-183
	10b	Important follow-up diagnostic and other test results	174-179, Fig 4-5-6
	10c	Intervention adherence and tolerability (How was this assessed?)	180-183
	10d	Adverse and unanticipated events	Not observed
Discussion	11a	A scientific discussion of the strengths AND limitations associated with this case report	285-304
	11b	Discussion of the relevant medical literature **with references**.	217-284
	11c	The scientific rationale for any conclusions (including assessment of possible causes)	214-237, 269-284
	11d	The primary "take-away" lessons of this case report (without references) in a one paragraph conclusion	313-321
Patient Perspective	12	The patient should share their perspective in one to two paragraphs on the treatment(s) they received	306-311
Informed Consent	13	Did the patient give informed consent? Please provide if requested	Yes ☒ No ☐
